# A case report of fatal disseminated fungal sepsis in a patient with ARDS and extracorporeal membrane oxygenation

**DOI:** 10.1186/s12871-020-01031-9

**Published:** 2020-05-07

**Authors:** Stefanie Prohaska, Philipp Henn, Svetlana Wenz, Leonie Frauenfeld, Peter Rosenberger, Helene A. Haeberle

**Affiliations:** 1 Department of Anesthesiology and Intensive Care Medicine, Intensivstation 39, Tübingen University Hospital, Eberhard-Karls-University, Hoppe-Seyler-Str. 3, 72076 Tübingen, Germany; 2 Department of Pathology, Tübingen University Hospital, Eberhard-Karls-University, Tübingen, Germany

**Keywords:** Mucormycosis, ARDS, ECMO

## Abstract

**Background:**

With the following report we want to present an unusual case of a patient suffering from acute respiratory distress syndrome with early discovery of bacterial pathogens in bronchoalveolar liquid samples that developed a fatal undiscovered disseminated fungal infection.

**Case presentation:**

A 67-year-old man was admitted to our university hospital with dyspnea. Progressive respiratory failure developed leading to admission to the intensive care unit, intubation and prone positioning was necessary. To ensure adequate oxygenation and lung protective ventilation veno-venous extracorporeal membrane oxygenation was established. Despite maximal therapy and adequate antiinfective therapy of all discovered pathogens the condition of the patient declined further and he deceased. Postmortem autopsy revealed Mucor and Aspergillus mycelium in multiple organs such as lung, heart and pancreas as the underlying cause of his deterioration and death.

**Conclusion:**

Routine screening re-evaluation of every infection is essential for adequate initiation and discontinuation of every antiinfective therapy. In cases with unexplained deterioration and unsuccessful sampling the possibility for diagnostic biopsies should be considered.

## Introduction

ARDS may be caused by a variety of conditions but in immunocompromised patients it is mainly due to infection. In this patient the pattern of infection by Pneumocystis jiroveci, *Staphylococcus aureus*/MSSA, *Candida dubliniensis*, Cytomegalovirus and *Legionella pneumophila* reflecst the compromised immune function. The mortality of immunosuppressed patients suffering ARDS is increased [1] regardless of the severity of the disease. Polimicrobial infections are seen regularly in immunocompromised critically ill patients. Fungal coinfections were described in children [2] and adults suffering ARDS [3, 4] due to viral infections. Pneumocystis jiroveci is often found in immunocompromised patients [5], as is the reactivation of Cytomegalovirus [6].

The differentiation of fungal contamination or infection in non-hematological patients may be challenging. Risk factors for fungal infections or coinfections in non-hematological ICU-patients are numerous, but not suitable as a distinguishing factor between infection and contamination. Diagnostics and first line treatment of the most common invasive fungal infections are listed in Table 1.

**Table 1:** most common invasive fungal infections [7] with additional diagnostics and first line treatment [8–11]

	Diagnostics		Treatment
Candida spp.	Direct microscopy and histopathology, cultur	Blood cultures B-D-Glucan Serum-Mannan/ anti-Mannan (in Candidaemia)	Echinocandins
Aspergillus spp.		CT (chest) Galactomannan (Serum, BAL)	Isacuconazol Voriconazol
Mucorales		CT (chest)	Surgical debridement Lipos. Amphotericin B Posaconazol (salvage treatment)

The reported single cases about mucormycosis increased lately. Most cases were described in patients with malignancies, organ transplantation, HIV or DM (recently reviewed in [12]). Recently Jiang and coworkers suggested the liquid-based cytopathology to identify mucorales promptly in samples obtained by bronchial brushes [13]. Much like with conventional cultures, the result may be difficult to interpret due to overgrowth.

## Case presentation

**Fig. 1:**
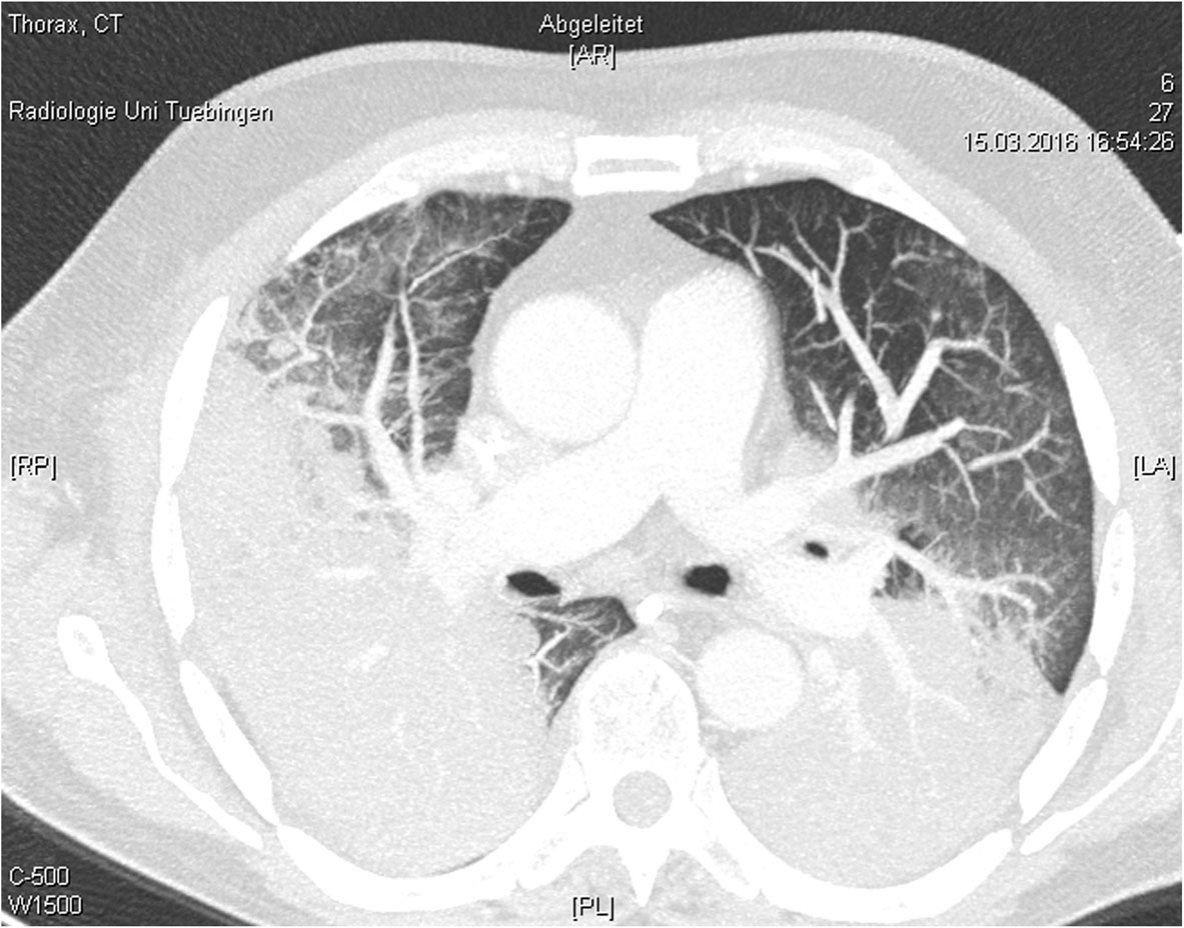
CT scan of the lung on day 1 after admission

A 67-year-old man with progressive dyspnea over 2 days was presented to the emergency department. Due to respiratory insufficiency he required intubation and initiation of mechanical ventilation and was therefore directly admitted to the ICU. The patient had a history of high-dose steroid therapy (dexamethasone 24 mg/day) for 5 weeks prior because of a spinal (suspected ependymoma presenting with spinal bleeding and paraplegia). His body temperature peaked at 40.4 °C approximately 2 h after admittance to the ICU. Leukocyte counts were normal but C-reactive protein (CRP) and Procalcitonin (PCT) levels were elevated (CRP 45.05 mg/ml, PCT 5.59 ng/ml). Several blood and BAL samples were taken for microbiological diagnosis. Anti-infective therapy was started with Piperacillin/Tazobactam and Clarithromycin in accordance with the local standard. On the following day CT scan showed bipulmonary infiltrates and no signs of pulmonary embolism (Fig. 1). At this point adequate ventilation required high driving pressures (paO_2_/FiO_2_ 77, pressure control ventilation, Pmax 32 mbar). Tidal volume goals were calculated with 6 ml/kg body weight. Prone positioning for about 19 h significantly improved the patient’s oxygenation and ventilation settings (paO_2_/FiO_2_ 207, pressure control ventilation, Pmax 24 mbar). On the second day we received the first results from the bronchoalveolar lavage. PCR for Pneumocystis jiroveci, Legionella species and Cytomegalovirus was positive. PCT levels peaked at 63.02 ng/ml and CRP levels at 56.18 mg/ml while leukocyte counts were remaining within normal range. Anti-infective therapy was changed to Primaquine, Clindamycin, Ganciclovir and Levofloxacin. Results from PCR and cultures 2 days later showed Pneumocystis jiroveci, *Staphylococcus aureus*/MSSA, *Candida dubliniensis*, Cytomegalovirus and *Legionella pneumophila*. Blood tests showed signs of a disseminated Cytomegalovirus infection with 13.800 copies per ml CMV DNA. Renal replacement therapy was started. On day 5 after admission to the ICU, the patient’s condition was rapidly deteriorating (FiO_2_/paO_2_ 50–60, pressure control ventilation, PEEP 14 mbar, Pmax 31, paCO_2_ 66, pH 7.12, BE − 7.8). Due to continuing severe septic shock (Lactat 8.3 mmol/l, Norepinephrine 1.5 μg/kg/min) and persistent risk of hypoxemia after interdisciplinary discussion extracorporal veno-venous oxygenation was established. In cases of septic shock extracorporal veno-arterial oxygenation is often limited due to higher heart time volumes in sepsis and developing harlequin phenomenon with insufficient systemic oxygenation. In these cases a veno-veno-arterial ECMO might be an option if the cardiac function is sufficient. After the start of veno-venous ECMO therapy, the patient stabilized slowly and the lactate levels decreased. There was no need for an additional arterial cannulation.

**Fig. 2:**
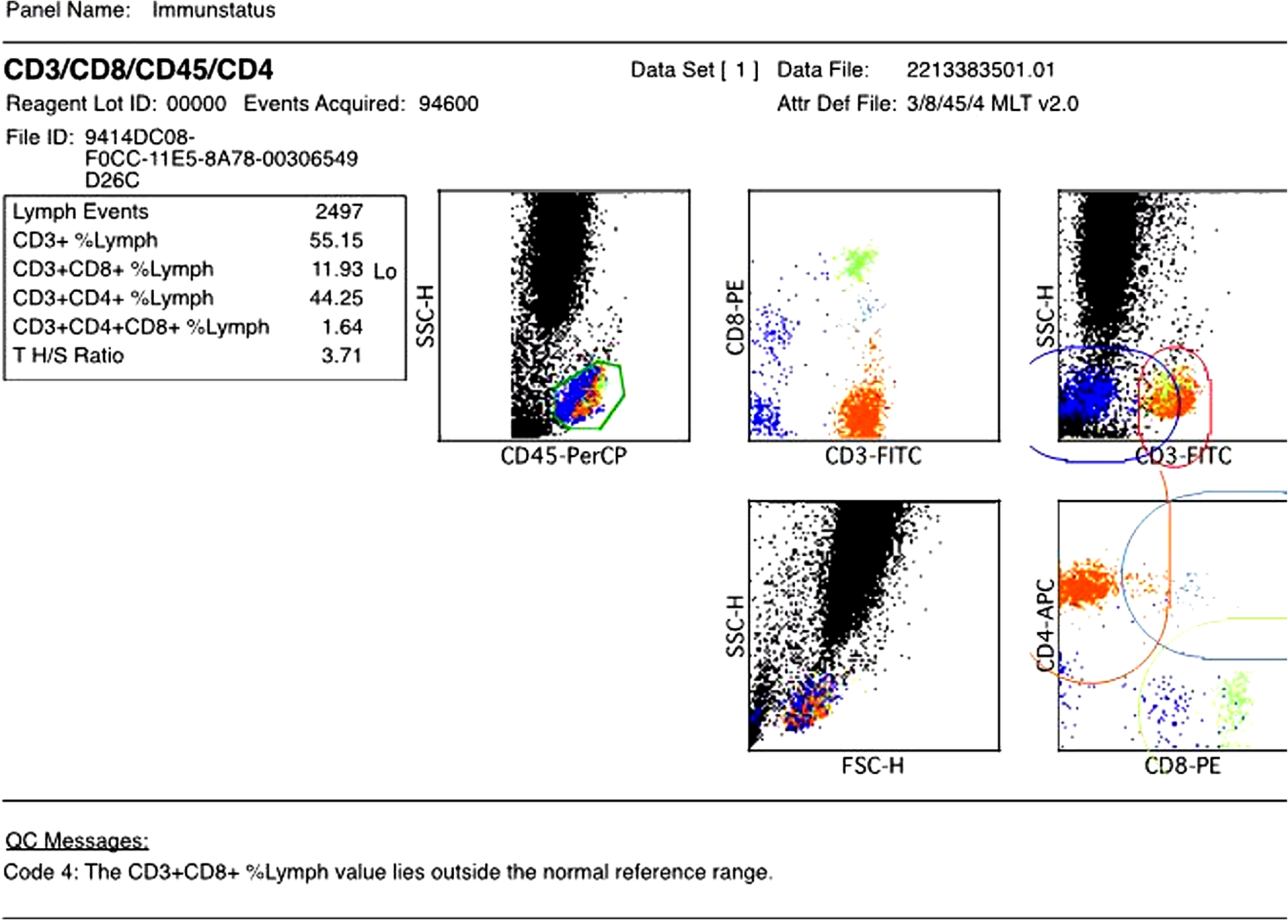
T-cell distribution on day 3 after admission

Anti-infective therapy was expanded to cover the detected and suspected pathogen spectrum: *Legionella pneumophila* (Azithromycin, Levofloxacin), *Staphylococcus aureus*/MSSA (Linezolid), *Pneumocystis jirovecii* (Trimethoprim/Sulfamethoxazole), *Candida dubliniensis* (Anidulafungin), Cytomegalovirus (Ganciclovir). In addition an antibody deficiency syndrome was treated with intravenous IgM-enriched immunoglobulin (Pentaglobin®) substitution. FACS analysis showed a decreased subset of T-suppressor cells (CD3 + CD8+/CD45+) and NK Lymphocytes (CD16 + 56+/CD45+) (Fig. 2). On day 6, signs of hepatic failure and disseminated intravascular coagulation were developing with rapidly declining platelet count and coagulation parameters, in spite of repeated transfusion and substitution. Lactate levels were rising and CT scan now showed massive bipulmonary infiltration with multiple pulmonary embolism and signs of kidney and cerebral ischemia due to disseminated embolism. This was primarily interpreted as a sign of disseminated intravascular coagulation but echocardiography was scheduled for the following day to rule out endocarditis and anticoagulation was switched from Heparin to Argatroban. Blood samples were sent to an extern laboratory to rule out HIT. Based on the impaired hemeostasis with severe thrombopenia (14.000/μl), biopsie on ECMO was abandoned and echocardiography postponed.

On day 7 after the initiation of anti-infective therapy *Candida glabrata*, Pneumocystis jiroveci, Cytomegalovirus and *Legionella pneumophila* were still present in BAL cultures. Even though MSSA was not detected anymore, Flucloxacillin was added to cover all bases and Anidulafungin was changed to Voriconazol.

**Fig. 3:**
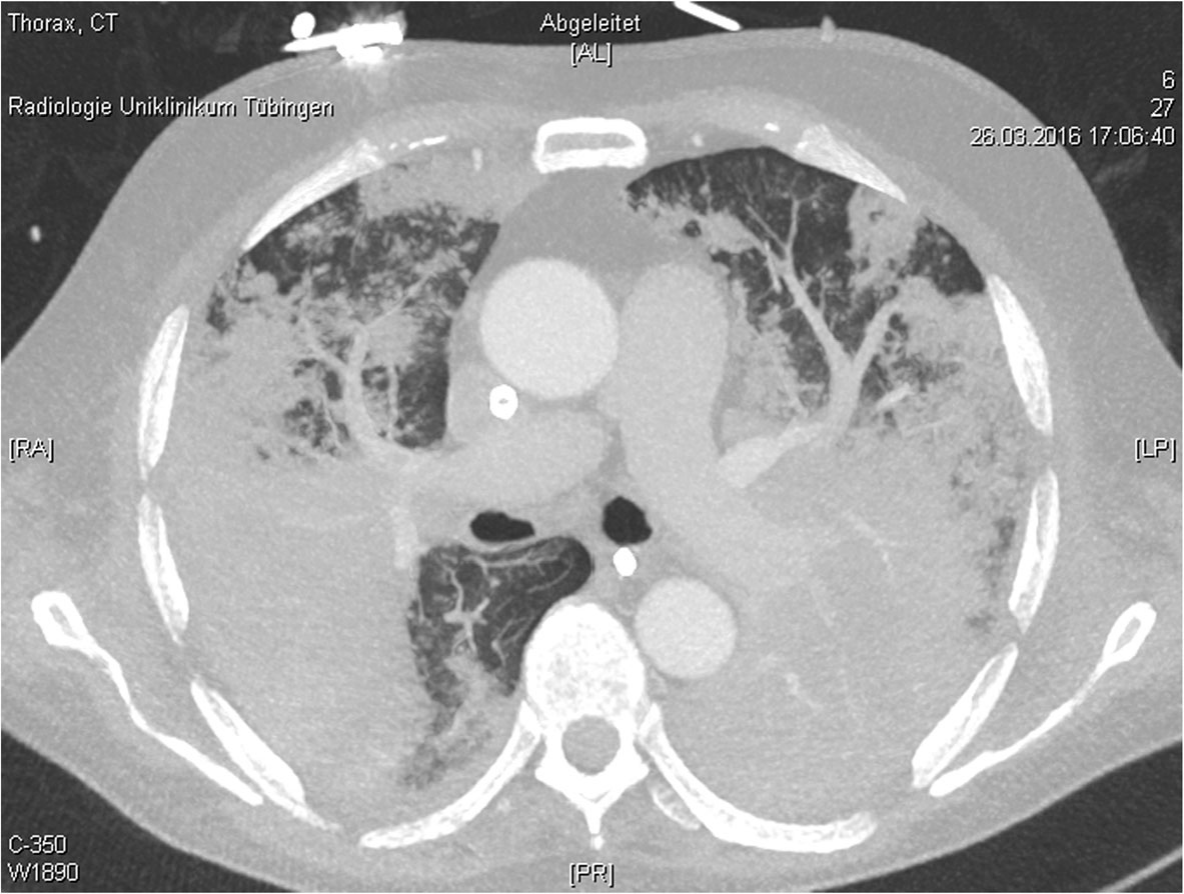
CT scan of the lung on day 12 after admission

Still, there were no signs of improvement. The signs of pulmonary, renal and hepatic failure and clinical signs of disseminated intravascular coagulation were still progressing. D-Dimers rose up to 42 μg/ml FEU. When the patient failed to awake after discontinuation of sedation we again performed CT scan on day 12. The massive bipulmonary infiltration was again progressing with signs of possible pulmonary hemorrhage (Fig. 3). The CT scan of the brain showed diffuse intracerebral hemorrhages with signs of increased intracranial pressure. Without further options and no achievable therapeutic goal extracorporeal membrane oxygenation was stopped. The patient died within minutes.

**Fig. 4:**
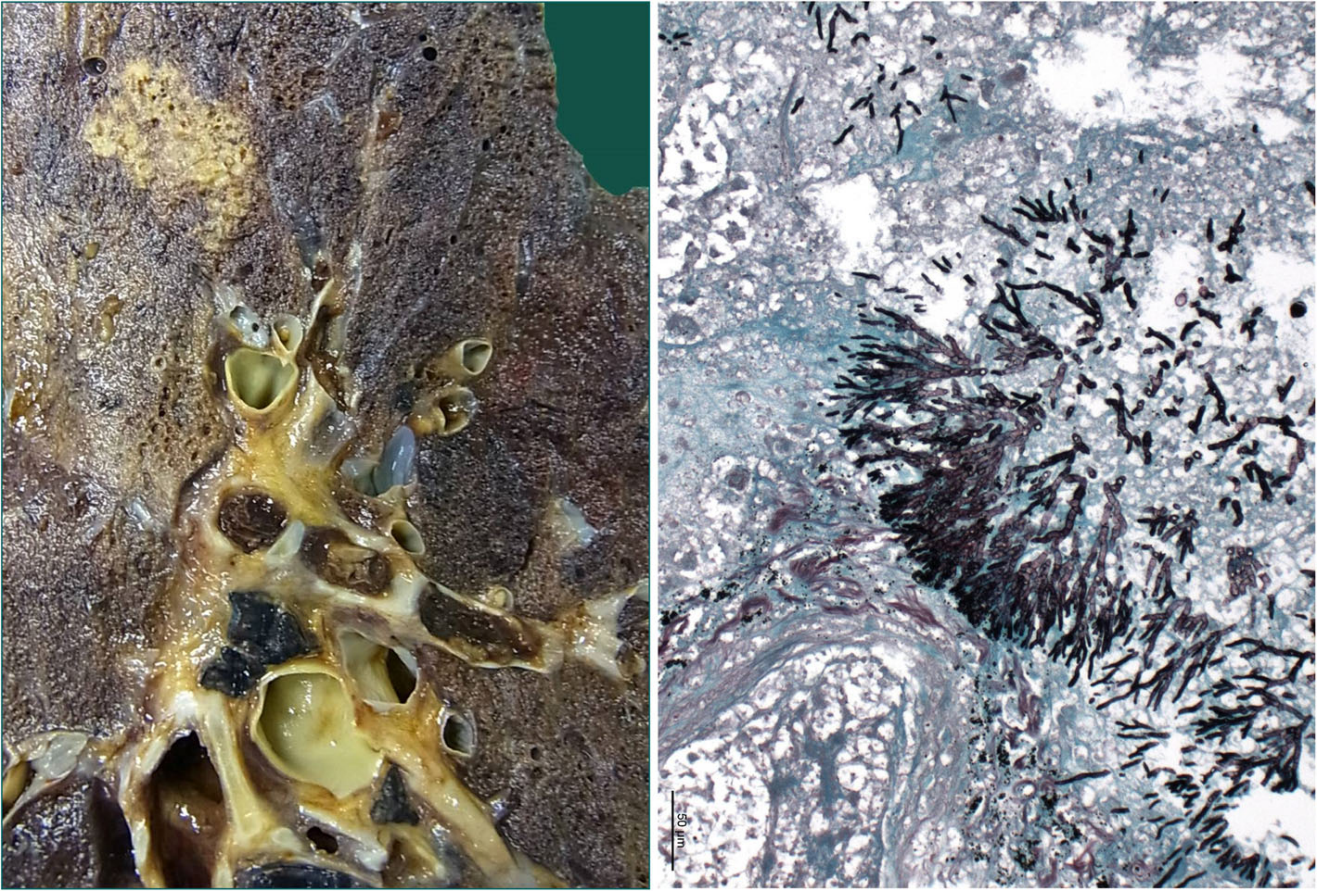
lung tissue, macroscopy (left) and Grocott-Gomori methenamine silver stain (right)

**Fig. 5:**
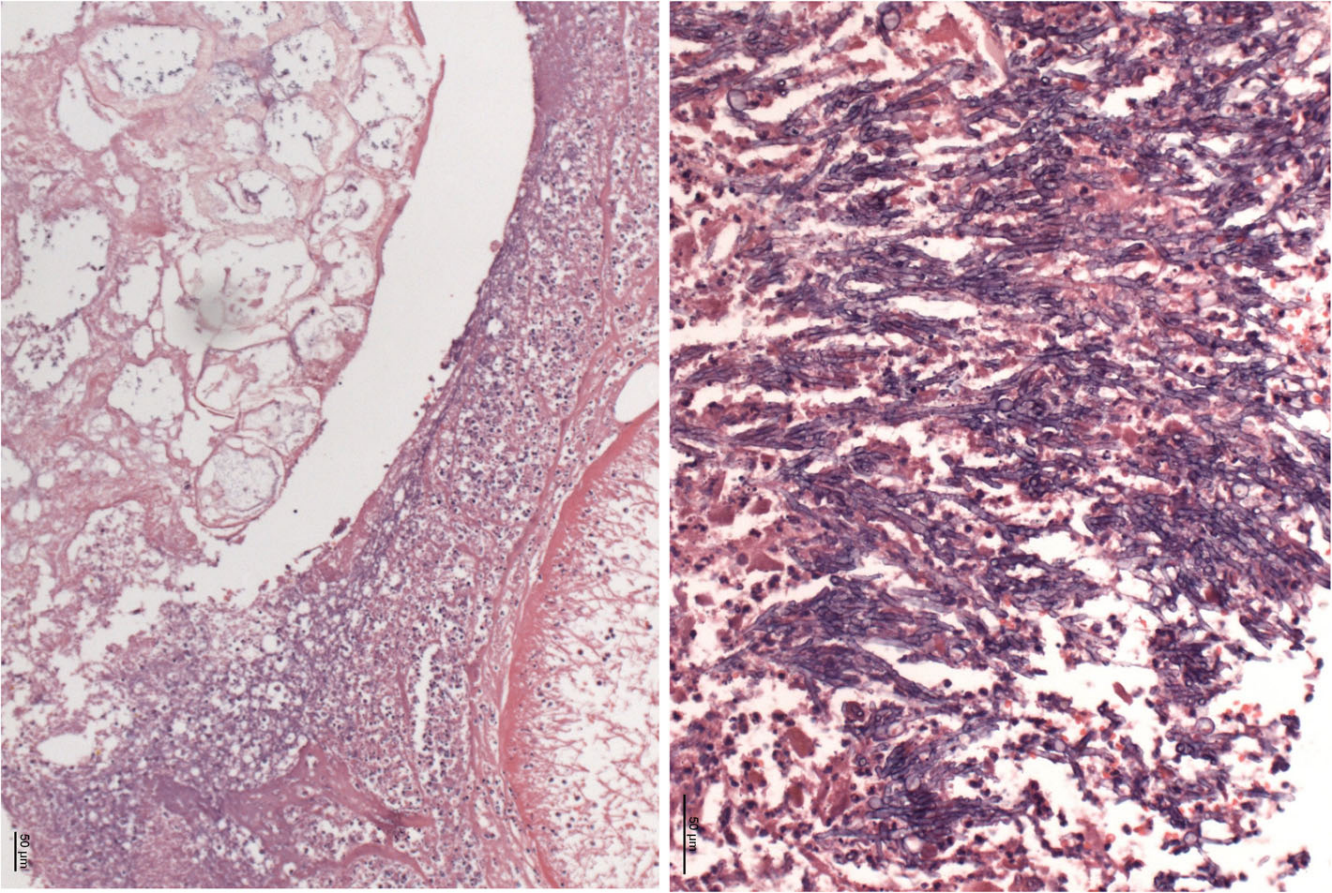
Pancreas tissue (left) and Myocard tissue (right)

The autopsy revealed the following findings:

1. Extensive intracerebral hemorrhage of both hemispheres, with emphasis of the left side, with cerebral edema and signs of hypoxic encephalopathy, as well as upper and lower herniation. No signs of fungal infiltration inside the brain.
2. Intramedullary malignant melanoma at the height of thoracic vertebra 1.
3. Massive infarct pneumonia on both sides. Lung parenchyma with evidence of Mucor and Aspergillus mycelium with angio-invasive/−destructive and focal bronchi-destructive growth. Focal parenchymatous hemorrhage on both sides. (Fig. 4)
4. Numerous infarctions (max. 0.5 cm) with focal Aspergillus and Mucor colonization in the myocardium. Accompanied by a very pronounced phlegmonous purulent myocarditis. (Fig. 5, right)
5. Kidneys: On both sides numerous infarctions (max. 1.5 cm) with Aspergillus and Mucor colonization with angio-invasive and glomeruli-destructing growth. Acute renal failure.
6. Multiple sharply delineated ulcer with raised margins and focal Aspergillus and Mucor colonization, predominantly in the corpus and antrum of the stomach, as well as in the whole colon.
7. Chronic recurrent pancreatitis with fatty necrosis. Several stray herds with Mucor and Aspergillus mycelium detection. (Fig. 5, left)
8. No fungus detection in paraaortal lymph nodes but aspergillus and Mucor colonization in the adjoining tissue.

## Discussion and conclusions

ARDS may be caused by a variety of conditions and mortality remains high. Even more so if the patient is immunocompromised due to medical therapy or infection. Fungal infections are hardly ever the first pathogens thought of, but fungal coinfections were described in children suffering ARDS due to viral infection [2]. Risk factors for fungal infections in ICU-patients are numerous, including immunosuppressive drugs and the differentiation of contamination or infection in non-hematological patients is challenging.

In this case the decision to deescalate and stop the therapy was based on the CT scan of the brain. The scan showed diffuse intracerebral hemorrhages with signs of increased intracranial pressure. The situation was evaluated and deemed to be infaust. With the knowledge of the autopsy findings, the overall situation of the mucor infection must now also be regarded as hopeless. Our initial discussion and decision to establish ECMO therapy was based upon the facts known at the time.

Although the reported single cases about mucormycosis increased lately, they are rare. Most cases were described in patients with malignancies, organ transplantation, HIV or DM (recently reviewed in [12]). Our patient was affected by none of these diseases but had received corticoid therapy and was therefore immunocrompromised.

There is some evidence, that mucormycosis and aspergillosis may be linked to previous antifungal therapy [14] although mainly in patients with hematological disorders. Therefore the onset of antifungal therapy is an important issue. Based on the expert opinion of the European Society of Anesthesia Intensive Care Scientific Subcommittee [15] the decision pathway in this case was correct. Initially the colonization index was < 0.5; candida score < 3. PCR- and Serum-tests were negative for fungal infection. 1,3-Beta-D-Glucan was not applied. However, the interpretation of this marker in patients infected by pneumocystis may be challenging [16]. The serological test (Platelia®; Bio Rad; München) did not prove positive Aspergillus-Galaktomannan-Antigen until 2 days before the patient’s death. In addition diagnosis of pulmonary mucormycosis by conventional culture may be difficult due to overgrowth. Microscopical examination of BAL may lead to misinterpretation due to contamination. Histopathological examination may be a valid option, although it is of risk in patients with anticoagulation and/or disseminated intravascular coagulation [8, 17]. Recently Jiang and coworkers suggested the liquid-based cytopathology to identify mucorales promptly in samples obtained by bronchial brushes, which could be a less invasive method to detect this infection promptly [13].

Considering and even re-considering frequent risk factors of fungal infections, e.g. mucormycosis and aspergillus, might be more fruitful than pursuing the question of how to provide evidence of the pathogen. There is no evidence but according to data obtained in the few hundred known cases of mucormycosis, history of poorly controlled diabetes in combination with impaired cell-mediated immune function including neutropenia are mainly the issue. Recent data suggests that T cells may play an important role in host defense to fungal disease [18, 19]. Like in our patient, lymphopenia may be an important indicator for the application of frequent fungal screening and fungal prophylaxis.

Routine screening before starting an antifungal prophylaxis and frequent re-evaluation of every infection are essential for adequate initiation and discontinuation of every fungal therapy especially with patients at high risk for fungal infections. All patients receiving immunosuppressive therapy, for whatever reason, must be included in this group. In case of assumed mucor infection the decision for biopsy should be taken into account for ARDS patients with progressive lung inflammation of unknown origin when all standard samples fail to provide an edaquate explanation for the patients status, since the risk to die due to mucor may outweigh the risk of fatal bleeding due to the biopsy. But this decision needs to be based on a detailed risk/benefit analysis for each patient.

## Data Availability

Additional clinical data is available on request. Please contact the corresponding author for any additional clinical data. This case report contains five figures. All figures have been uploaded with the manuscript.

## Abbreviations

ARDS: Acute respiratory distress syndrome
BAL: Bronchoalveolar lavage
CMV: Cytomegalovirus
DNA: Desoryribonucleic acid
CRP: C-reactive protein
CT: Computed tomography
DIC: Disseminated intravascular coagulation
DM: Diabetes mellitus
ECMO: Extracorporeal membrane oxygenation
FACS: Fluorescence activated cell sorting
FiO_2_: Fraction of inspired oxygen
paO_2_: Arterial partial pressure of oxygen
HIT: Heparin induced thrombocytopenia
HIV: Human immunodeficiency virus
ICU: Intensive care unit
IgM: Immunoglobulin M
INR: International normalized ratio
MSSA: Methicillin sensitive *Staphylococcus aureus*
PCR: Polymerase chain reaction
PCT: Procalcitonin
Pmax: Peak pressure
